# High-Level Fluorescence Labeling of Gram-Positive Pathogens

**DOI:** 10.1371/journal.pone.0019822

**Published:** 2011-06-22

**Authors:** Simone Aymanns, Stefanie Mauerer, Ger van Zandbergen, Christiane Wolz, Barbara Spellerberg

**Affiliations:** 1 Institute of Medical Microbiology and Hygiene, University of Ulm, Ulm, Germany; 2 Interfakultäres Institut für Mikrobiologie und Infektionsmedizin Eberhard-Karls-Universität, Tübingen, Germany; National Institutes of Health, United States of America

## Abstract

Fluorescence labeling of bacterial pathogens has a broad range of interesting applications including the observation of living bacteria within host cells. We constructed a novel vector based on the *E. coli* streptococcal shuttle plasmid pAT28 that can propagate in numerous bacterial species from different genera. The plasmid harbors a promoterless copy of the green fluorescent variant gene *egfp* under the control of the CAMP-factor gene (*cfb*) promoter of *Streptococcus agalactiae* and was designated pBSU101. Upon transfer of the plasmid into streptococci, the bacteria show a distinct and easily detectable fluorescence using a standard fluorescence microscope and quantification by FACS-analysis demonstrated values that were 10–50 times increased over the respective controls. To assess the suitability of the construct for high efficiency fluorescence labeling in different gram-positive pathogens, numerous species were transformed. We successfully labeled *Streptococcus pyogenes*, *Streptococcus agalactiae*, *Streptococcus dysgalactiae* subsp. *equisimilis*, *Enterococcus faecalis*, *Enterococcus faecium*, *Streptococcus mutans, Streptococcus anginosus* and *Staphylococcus aureus* strains utilizing the EGFP reporter plasmid pBSU101. In all of these species the presence of the *cfb* promoter construct resulted in high-level EGFP expression that could be further increased by growing the streptococcal and enterococcal cultures under high oxygen conditions through continuous aeration.

## Introduction

Green fluorescent protein (GFP) of the jellyfish *Aequorea victoria* is an excellent fluorescent marker since it can be expressed in heterologous hosts without the need for cofactors or specific substrates. It shines bright green if activated by blue or UV light [1] [2]. Successful expression of GFP activity has been shown for numerous organisms ranging from bacteria to mammalian cells of diverse tissue types [3] [4] [5] [6]. EGFP (enhanced green fluorescent protein), is a GFP variant causing a greatly increased fluorescence intensity compared to the GFP wildtype protein [7]. In contrast to wildtype GFP, which to some extend is found inactive in inclusion bodies, the solubility of EGFP is considerably enhanced [7]. GFP variants are very stable molecules [8] [9] and can be used without providing evidence of harmful effects on living cells [10] [11] [1]. Highest fluorescence values are observed in well oxygenated cultures of a pH-value of 7. The chromophore is activated at high oxygen conditions [12]. In alkaline as well as acidic conditions, the fluorescence intensity is noticeably reduced [13] [14].

Successful expression of GFP in different lactic acid bacteria has been reported from several laboratories [15] [16] [13]. The ability of streptococcal strains expressing GFP to survive *in vivo* and to be suitable for pathogenesis studies has been demonstrated for *Streptococcus suis* [15]. Streptococci are lactic acid bacteria that comprise numerous facultative pathogenic species and several highly virulent bacterial pathogens, like *S. pyogenes*. They are facultative anaerobes that grow well under low oxygen conditions often requiring 5% CO_2_ for optimal growth. Together with an increased tolerance for low pH values these physiological features are in contrast to the conditions under which optimal GFP expression is observed. These facts may explain why the majority of GFP- constructs in streptococci so far resulted in measurable but moderate fluorescence values [17] [18] that could not match the levels of expression observed in gram-negative bacteria like *E. coli*.

In the present study, a vector construct that was derived from plasmid pAT28 [19] and contains a promoterless copy of the *egfp* gene downstream of its multiple cloning site was studied. In the specific construct the expression of EGFP is driven the promoter of the CAMP-factor gene of *S. agalactiae*. The CAMP-factor is a cell surface associated molecule that is responsible for the cohemolysis of erythrocytes in the presence of *S. aureus* and independent from the *S. agalactiae* ß-hemolysin. This phenomenon is often used for diagnostic purposes in the species identification of *S. agalactiae*. The identification of the gene *cfb*, encoding the CAMP-factor, was published several years ago and includes the description of the promoter region [20]. The CAMP-factor has been shown to represent a pore forming toxin in *S. agalactiae* [21]. A gene similar to *cfb* is present in *S. pyogenes* [22] (designated *cfa*), indicating that the function of the *cfb* promoter may not be limited to *S. agalactiae.* To assess the potential role of the plasmid as a general tool to provide EGFP-labeling, the construct was investigated for its ability to enable high-level EGFP expression in numerous gram-positive hosts.

## Materials and Methods

### Bacterial strains, cell line and growth conditions

The bacterial strains and plasmids used in this study are listed in Table 1. Gram positive bacteria were grown at 37°C in THY (Todd Hewitt Broth (Oxoid, Wesel, Germany)) supplemented with 0.5% yeast extract) containing 120 mg/l spectinomycin. The monocytic cell line THP-1 (ATCC, East Greenwich, RI, USA) was grown at a density of 3×10^5^ cells/ml at 37°C in a humidified atmosphere containing 5% CO_2_ in complete medium (RPMI 1640 medium (Sigma, Deisenhofen, Germany), supplemented with 10% heat inactivated FCS, 50 µM 2-Mercaptoethanol, 2 mM L-glutamine, 10 mM Hepes, 100 µg/ml penicillin and 160 µg/ml gentamicin, all Seromed-Biochrom (Berlin, Germany). Cells were passaged every 72 h. In order to differentiate THP-1 cells into macrophages, cells were cultured for 24 h at 37°C and 5% CO_2_ in complete medium supplemented with 10 ng/ml Phorbol 12-myristate 13-acetate (PMA, Sigma, Deisenhofen, Germany)

**Table 1:** Bacterial strains and plasmids.

Strains	Description	Source or reference
*E. faecalis* BSU 386	*Clinical isolate*	Ulm collection
*E. faecium* BSU 385	*Clinical isolate*	Ulm collection
*S. anginosus* BSU 458	*ATCC 12395*	American Type Culture Collection
*S. mutans* BSU 269	*DSM 20523*	DSMZ collection
*S. pyogenes* BSU 317	clinical isolate	Ulm collection
*S.agalactiae* BSU6 serotype Ia strain	clinical isolate	Ulm collection
*S. dysgalactiae* subsp. *equisimilis* group C BSU 225	Clinical isolate	Ulm collection
*S. dysgalactiae* subsp. *equisimilis* group G BSU 263	*AC 1140*	Aachen collection
*Staphylococcus aureus* BSU 542	RN 4220	Kreiswirth et al., 1983
*Escherichia coli* DH5α	*endA1 hsdR17 supE44* Δ*lacU169 (*φ*80lacZDM15) recA1 gyrA96 thi-1 relA1*	Boehringer Ingelheim
**Plasmids**		
pAT28	Spec^r^ ori pUC ori pAmβ1	Trieu-Cuot et al., 1990
pEGFP	Am^r^, source of *egfp*	Clontech Laboratories
pBSU100	pAT28 derivative carrying *egfp*	this study
pBSU101	pAT28 derivative carrying *egfp* under the control of the *cfb* promoter	this study

### Construction of the reporter plasmid pBSU101

**Figure 1:**
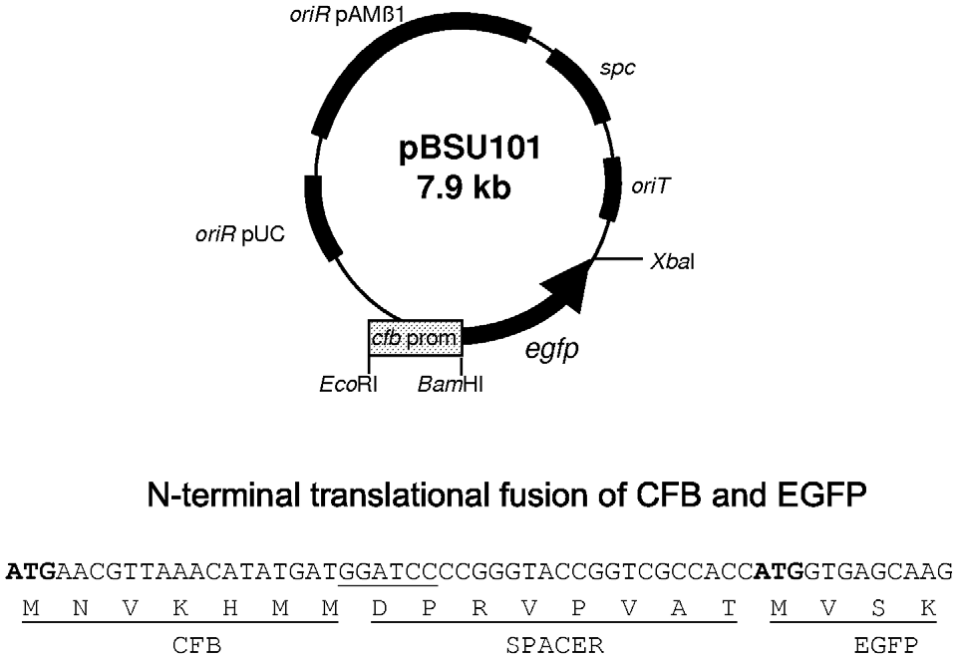
Depicted is a vector map of shuttle vector pBSU101 that was used in this study to label different gram-positive bacteria with EGFP. The multiple cloning site (MCS), the different origins of replication (for use in gram-positive hosts, *oriR* pAMß1, and gram-negative bacteria, *oriR* pUC) and the spectinomycin (*spc*) resistance gene are indicated. The plasmid is a derivative of pAT28. Expression of EGFP in pBSU101 is controlled via the promoter region of the CAMP-factor gene *cfb* of *Streptococcus agalactiae*. The plasmid carries the promoter region and the ATG startcodon of *cfb*, resulting in a translational fusion of the first 7 amino acids of CFB to the N-terminus of EGFP as depicted. Between the CFB fragment and the start of EGFP a small spacer of 8 amino acids is found that does not appear to disturb efficient EGFP expression.

The plasmids pBSU101 and pBSU100 were constructed in *E. coli* DH5α. Both plasmids are derivatives of pAT28. A promoterless copy of the *egfp* gene was inserted into pAT28 via the *Xba*I restriction sites flanking this gene in the vector pEGFP (BD Biosciences, San Jose, CA), which served as a source for *egfp*. Correct insertion of *egfp* in this construct was confirmed by DNA sequencing. The resulting plasmid was designated pBSU100 and served as control vector for subsequent experiments. To control expression of EGFP in pBSU101 the promoter region of the *Streptococcus agalactiae* gene *cfb* was inserted into pBSU100. The promoter region including the ATG startcodon of *cfb* was amplified by PCR using the primers cfbProm1 and cfbProm2 (Table 2) which add *Eco*RI and *Bam*HI restriction sites to the *cfb* promoter. Following digestion of the PCR product and the pBSU100 vector with *Bam*HI and *Eco*RI, plasmid and insert were ligated applying standard molecular biology techniques. Selection was carried out on LB (Luria Bertani) agar plates supplemented with 125 mg/l of spectinomycin. Correct insertion was confirmed by performing a PCR reaction using primers PBSU100-for1 and PBSU100-rev1, which are flanking the multiple cloning site of pBSU100 (see Table 2) and subsequent sequencing of the PCR product. The insertion of the fragment into pBSU100 resulted in a translational fusion of the first 7 amino acids of CFB to the N-terminus of EGFP as depicted in Fig. 1.

**Table 2:** Primers used for the construction and analysis of pBSU100, pBSU101.

name	sequence	target gene
*cfb*-Prom1	5′- AAC GAA TTC ATC TAA AAT AGT ACG C-3′	*cfb* of *S. agalactiae*
*cfb*-Prom2	5′- CGC GGA TCC ATC ATA TGT TTA ACG-3′	*cfb* of *S. agalactiae*
PBSU100-for1	5′-GTT GTG TGG AAT TGT GAG CGG-3′	MCS pBSU100, pBSU101
PBSU100-rev1	5′-CCT TGA AGA AGA TGG TGC GC-3′	MCS pBSU100, pBSU101

### Plasmid transformation

Transformation of the different bacterial strains was achieved by electroporation. For transformation of enterococcal and streptococcal strains the protocols of Friesenegger, *et al*. [23] and Ricci *et al*. were used [24] . The method relies on the generation of electrocompetent cells through repeated washes in 10% glycerol. For *S. aureus* the transformation was conducted as described in [25] using the restriction deficient strain RN4220 [26]. Recombinant streptococcal and enterococcal strains were selected on THB (Todd Hewitt broth) or blood agar plates supplemented with 120 mg/l spectinomycin.

### FACS analysis of EGFP expressing bacterial strains

Streptococal, enterococcal strains and *S. aureus* were grown overnight in THY broth at 5%CO_2_ or in THY broth under continuous shaking. Bacteria were harvested by centrifugation, washed once and resuspended in PBS (phosphate buffered saline, pH 7.4). Quantification of fluorescence was achieved by FACS analysis (FACSCalibur®, Becton Dickinson Immunocytometry Systems, San Jose, CA) using the following instrument settings: FSC: E00, SSC: 400, FL-1: 700. Relative fluorescence values were determined by analyzing 10 000 events for each sample.

### THP-1 experiments

Bacterial strains were grown overnight on 5% sheep blood agar plates supplemented by 125 mg/l of spectinomycin. Bacteria were resuspended in prewarmed RPMI (Sigma, Deisenhofen, Germany) without supplements, to a density of 10^7^ bacteria/ ml. 2×10^5^ THP-1 cells were incubated with the *S. agalactiae* strains at a multiplicity of infection of 1, 10, and 100 and kept for one hour in complete medium supplemented with 5% human plasma (Biochrom, Berlin, Germany) at 37°C and 5% CO_2_. Microscopy of THP-1 cells was performed with a Zeiss Axioskop-2® fluorescence microscope fitted with a Axiocam HR camera and Axiovison software version 4.6. For quantification samples were analyzed using a FACSCalibur® with CellQuest-Pro® software (Becton Dickinson, San Diego, CA).

## Results

### Establishment of the EGFP-construct in different streptococci and related genera

**Figure 2:**
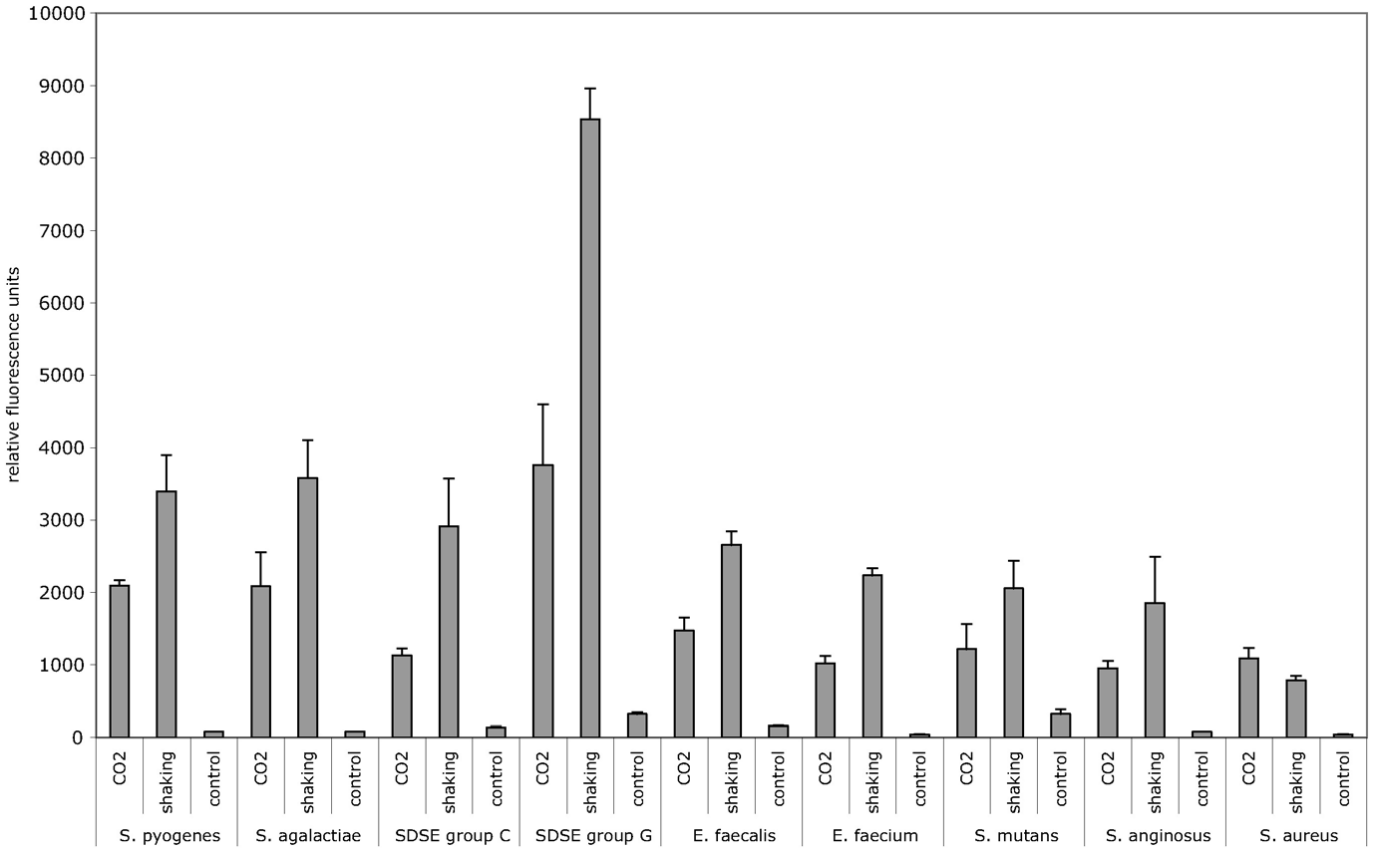
EGFP expression in different bacterial hosts was determined by FACS analysis. Streptococcal, enterococcal strains and *S. aureus* were grown overnight in THY broth as described in Material and Methods. Depicted are the values obtained for the ß-hemolytic species *S. agalactiae*, *S. pyogenes*, *S. dysgalactiae* subsp. *equsimilis* (SDSE) serogroup C, SDSE serogroup G, *Staphylococcus aureus*, the enterococcal species *E. faecalis* and *E. faecium* and the viridans group streptococci *S. mutans* and *S. anginosus*. Shown are mean values and standard deviations of five independent experiments.

**Figure 3:**
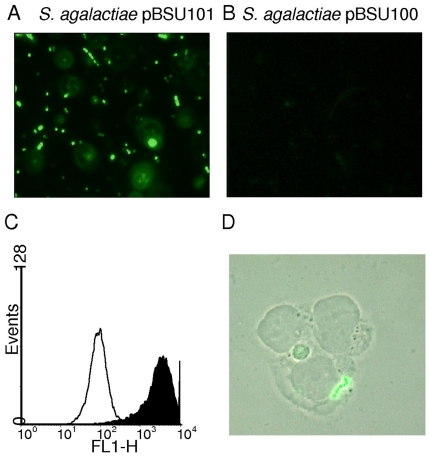
Fluorescence microscopy of *S. agalactiae* strain BSU6 transformed with pBSU101 (A) and pBSU100 as control (B). C: Histogramm (events versus FLH-1) of a FACS analysis of BSU6 harboring pBSU101 (black area) and pBSU100 (white area) D: THP-1 host cells were incubated with *S. agalactiae* strain BSU6 carrying plasmid pBSU101 as described in Materials and Methods for 1 hour and visualized with a Zeiss Axioskop-2® fluorescence microscope.

The reporter construct used in the present study is a derivative of the pAT28 plasmid [19]. This plasmid is a streptococcal *E. coli* shuttle vector present in multiple copies in the cytoplasm of transformed strains. A promoterless copy of *egfp* was integrated into pAT28 and expression of EGFP in this vector is driven by the promoter region of *cfb*, the CAMP-factor gene of *S. agalactiae* (Fig. 1). A translational fusion of the first seven amino acids of CFB to the N-terminal region of EGFP was created for optimal expression of EGFP. The construct was designated pBSU101 and resulted in brightly fluorescent *S. agalactiae* cells (Fig. 2), while control strains carrying the construct without promoter (pBSU100), did not reveal elevated fluorescence (Fig. 2, Fig. 3). To evaluate, if a similar expression of EGFP is achieved in other gram-positive hosts, pBSU101 was transformed successfully in numerous bacterial pathogens. Numerous streptococcal species including highly pathogenic ß-hemolytic strains (*S. pyogenes*, *S. dysgalactiae* subsp. *equisimilis*, and *S. agalactiae*) as well as viridans streptococci (*S. mutans*, *S. anginosus*) were used. To assess expression in related genera, enterococci (*Enterococcus faecalis* and *Enterococcus faecium*) and *Staphylococcus aureus* were transformed with the construct. In every bacterial species and strain that could be successfully transformed a bright fluorescence was detectable (Fig. 2). Unfortunately, despite repeated efforts to establish the vector construct in *S. pneumoniae*, transformation of this species with the pAT derived plasmid could not be achieved (Simone Bergmann, Braunschweig, personal communication), even though successful labeling of *S. pneumoniae* by GFP has been described [27].

### Level of EGFP-expression

**Figure 4:**
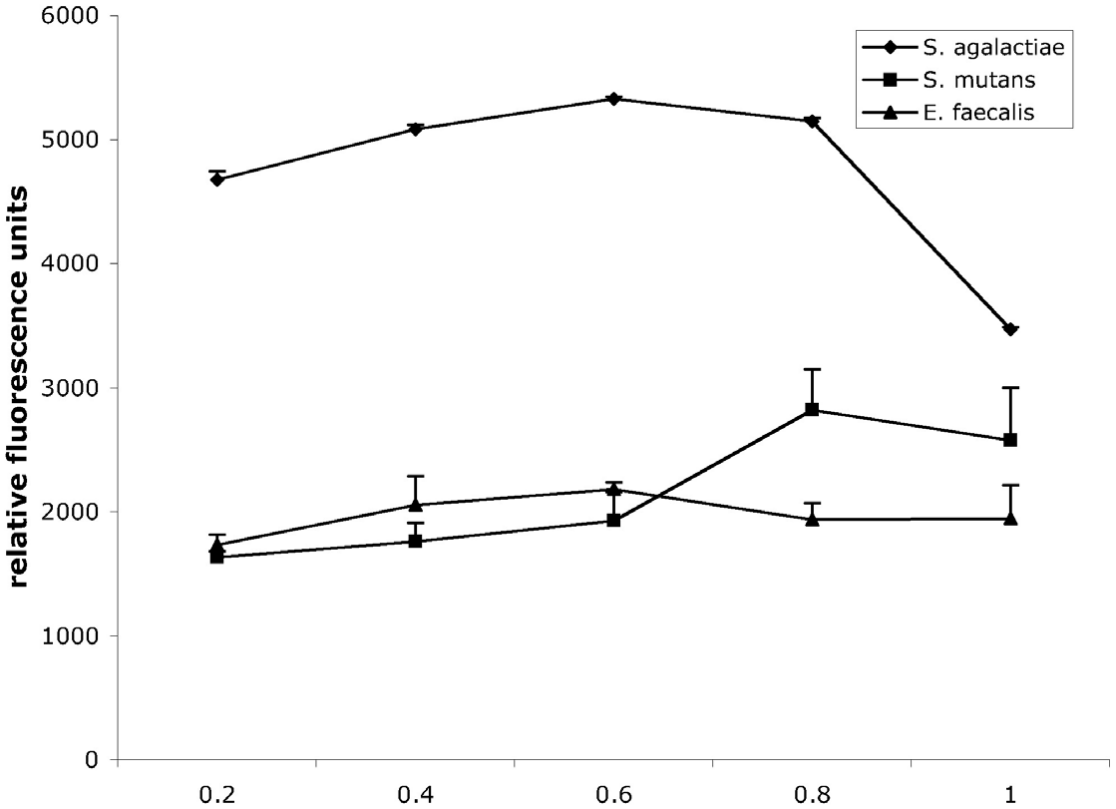
EGFP expression in different growth phases was measured by FACS analysis in *S. agalactiae, S. mutans,* and *E. faecalis*. Shown are relative fluorescence units obtained for bacteria grown to an optical density (OD) of 0.2, 0.4, 0.6 0.8 and 1.0. The values represent mean values and standard deviations of three independent experiments.

Due to the high fluorescence values that were observed after transfer of the plasmid, positive clones could easily be detected in a standard fluorescence microscope (Fig. 3). Quantification of the relative fluorescence in comparison to the negative control strains was performed by FACS analysis of overnight cultures in THY broth. In these measurements, an increase of 10 to 50 times over the baseline values that were observed for the control strains (Fig. 2) was noted. Typically relative fluorescence values around 1000–5000 could be measured while the negative control strains are around 100. Streptococci and enterococci are facultative anaerobes and often grow best under low oxygen conditions, which is not optimal for EGFP-folding. To assess the effect of providing the cultures with additional oxygen, we compared standard growth conditions with cultures grown under continuously shaking conditions. For all of the streptococcal and enterococcal strains, fluorescence intensity was higher in the agitated samples; increases between 65 and 125% were observed (Fig. 2). In contrast to these observations, growing *Staphylococcus aureus* under continuously shaking conditions had no effect (Fig. 2). Since EGFP expression in *S. aureus* appeared somewhat lower than in most streptococcal or enterococcal species (Fig. 2) we performed Western blot experiments with antibodies specific for EGFP to evaluate EGFP expression in *S. aureus*. However no indication for a degradation of EGFP could be observed in the immunoblot (data not shown). To investigate the level of EGFP expression provided by pBSU101 in different growth phases, cultures grown to an OD_(600 nm)_ of 0.2, 0.4, 0.6, 0.8, and 1.0 were analyzed by FACS. For these experiments three different bacterial species (*S. agalactiae*, *S. mutans* and *E. faecalis*) were selected representing ß-hemolytic streptococci, viridans streptococci and enterococci. In all of these species high-level EGFP expression can already be observed in the early logarithmic growth phase at an OD of 0.2 (Fig. 4).

### Plasmid stability

**Figure 5:**
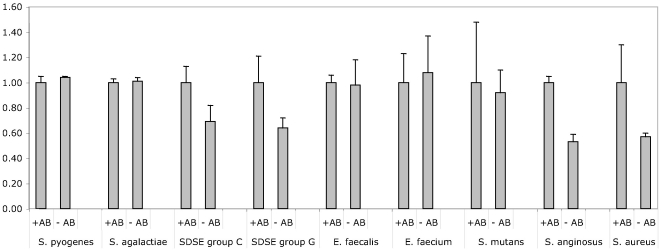
EGFP fluorescence in bacterial strains harboring pBSU101 following overnight growth in the presence (+AB) or absence (−AB) of antibiotic pressure was determined by FACS analysis. Fluorescence values obtained for each bacterial species following overnight growth under antibiotic pressure were set to a value of 1. Measurements were carried out for *S. agalactiae*, *S. pyogenes*, *S. dysgalactiae* subsp. *equsimilis* (SDSE) serogroup C, SDSE serogroup G, *E. faecalis*, *E. faecium*, *S. mutans, S. anginosus*, and *Staphylococcus aureus*. Shown are the results of three independent experiments and standard deviations.

Transformation by pBSU101 typically requires growth of the labeled bacteria under antibiotic pressure to ensure a stable propagation of the plasmid. To address the question if the plasmid is maintained in the absence of antibiotic pressure, bacterial strains harboring the plasmid were grown to saturation in liquid culture containing antibiotics, diluted 1∶100 in fresh medium and grown overnight in the presence and absence of spectinomycin supplementation. In all of the strains fluorescence values well above the negative controls could be observed, even after overnight growth without antibiotics. However, as to be expected, the plasmid was not completely stable in all of the strains. While no significant differences in the fluorescence values could be observed for *S. agalactiae E. faecalis, E. faecium* and *S. pyogenes* (Fig. 5). A marked drop of the fluorescence values in the bacterial cultures lacking antibiotics was seen for *S. dysgalactiae* subsp. *equisimilis* (group C and group G strains), *S. mutans*, *S. anginosus* and *Staphylococcus aureus.*

### Detection of eukaryotic host cells harboring EGFP-positive streptococci

**Figure 6:**
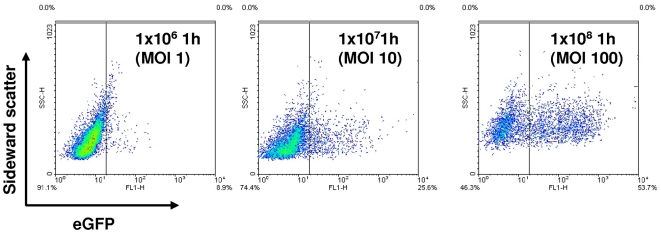
THP-1 cells were infected with *S. agalactiae* strain BSU6 carrying the plasmid pBSU101 at a multiplicity of infection (MOI) of 1, 10 and 100. After 1 hour of incubation detection of fluorescent THP-1 cells was performed by FACS analysis counting 10000 events. Depicted are the measurements for sidewardscatter and green fluorescence.

The suitability of the vector construct to visualize macrophage uptake of fluorescently labeled bacteria was investigated using the eukaryotic host cell line THP-1. PMA differentiated THP-1 cells are a model for human macrophages. THP-1 macrophages carrying the EGFP expressing *S. agalactiae* strain could easily be detected after 60 min of co-incubation (Fig. 3D). In addition infection experiments using macrophages and pBSU101 labeled *S. agalactiae* strain demonstrate a dose dependent increase of fluorescent THP-1 cells as assessed by FACS analysis (Fig. 6).

## Discussion

Following the successful construction of an EGFP-plasmid for *S. agalactiae,* we were interested to evaluate this plasmid as a general tool for other streptococcal species and related genera. It was possible to establish pBSU101 in a large variety of gram-positive species comprising different streptococci as well as enterococci and even *S. aureus*. Since transformation by pAT vectors has already been described for the genera *Bacillus* [28] and *Listeria* [29], including a pAT vector derivative carrying GFP for use in *Listeria* [30], it may very well be possible that the range of potential bacterial hosts for this plasmid is much broader.

The level of fluorescence that was provided by the integration of the *cfb* promoter to control *egfp* transcription in pBSU101 was unexpected. We had previously noted that the CAMP-factor gene of *S. agalactiae* appears to be well transcribed since visualization of *cfb* mRNA in Northern blot experiments required considerable lower amounts of total RNA than other *S. agalactiae* genes [31]. This observation is consistent with the finding that unexpected high levels of EGFP expression for streptococci were observed in our plasmid construct. The increase of relative fluorescence values (RFU) from a baseline of around 100 RFU or less to 1000–5000 RFU under the control of the *cfb* promoter was exceptional, if we compare it to other streptococcal promoters. In a recent publication [32] we examined the induction of the streptococcal C5a peptidase gene (*scpB*) in a derivative of pBSU100 with a slightly modified multiple cloning site. In this construct EGFP expression was controlled by the *scpB* promoter and generated under inducing conditions maximal relative fluorescence values of 200–250 RFU; a finding that clearly illustrates the strength of the *cfb* promoter. Several years ago a close homologue of the CAMP-factor, was discovered in *S. pyogenes* [22], an indication that it may be possible to use the promoter in other streptococcal species and related genera. Luckily the *cfb* promoter did not only work for many different gram-positive bacteria, but also resulted in continuous high-level expression of EGFP. We were not able to isolate any bacterial clones harboring pBSU101 but failing to demonstrate a strong increase in EGFP-expression. This finding may indicate that the promoter can also be used as a tool to induce hyperexpression of other genes in gram-positive hosts. However despite the strong expression of EGFP under the control of the *cfb* promoter, Fluorescence measurements that were obtained in the different bacterial species following transformation by pBSU101 demonstrated a considerable amount of variation (Fig. 2). Values between 1000 to 5000 RFU were noted. The reason for this heterogeneity is presently unclear and needs to be addressed in future investigations. Different copy rates of the plasmid, variations in the transcription or translation rates, degradation of EGFP in some species or variations in the activation of the chromophore may be responsible for the observed differences.

The remarkable high-level expression of EGFP under the control of the *cfb* promoter may also explain why the construct is able to overcome the less than favorable conditions for EGFP expression present in streptococci. Adequate oxygen levels are needed for GFP fluorophore formation [12] [33] [13] and constitute a problem regarding facultative anaerobic bacteria like streptococci and enterococci. These species often grow much better under decreased oxygen conditions. The low intracellular oxygen conditions have long been regarded as the reason why many GFP-constructs, that were used in streptococci so far, did only generate moderate fluorescence levels. That the oxygen level does indeed limit EGFP-expression in streptococci and enterococci is nicely shown by the substantial increase of fluorescence that was observed under continuous aeration of the growing cultures (Fig. 2). It has however to be kept in mind that while this is a very easy method to increase the fluorescence, it may not represent a truly physiologic condition for streptococci and may thus impair other bacterial functions.

Fluorescence labeling of bacterial pathogens for the investigation of microbial host cell interactions has been used for many years. One of the most commonly used substrates for this purpose is fluorescein isothiocyanate (FITC), which has also been used for many different applications in streptococci [34] [35]. However, FITC is covalently linked to bacterial surface proteins that may be important for the host pathogen interactions under investigation. Not surprisingly it has been demonstrated that FITC-labeling may affect the viability of microorganisms, impair the integrity of virulence factors and thus alter the pathophysiology of bacterial pathogens [11]. Fluorescence labeling by expression of GFP or its variants appears to be considerably less disruptive and much better suited for *in vivo* studies. One major advantage is that the intensity of the fluorescence label does not decrease with multiplication of the labeled microorganisms, rendering it a perfect tool for *in vitro* as well as *in vivo* investigations. In our experiments employing THP-1 cells we could already show that the detection of cell associated streptococci using FACS analysis as well as microscopic analysis works well. For many investigations of bacterial host interactions bacteria grown to early mid- or late logarithmic growth phase are used. Analysis of fluorescence values conferred by this plasmid in different growth phases shows that the high-level EGFP expression is already present at the early logarithmic growth phase of an OD of 0.2 (Fig. 4). A feature that is certainly interesting for researchers who need to fluorescently label bacteria in a logarithmic growth phase for their experiments. Since not in all experimental settings it is possible to maintain antibiotic pressure, we investigated the stability of the plasmid following removal of antibiotic pressure. As to be expected, plasmid loss does occur under these conditions. However after 24 hours of overnight growth in the absence of antibiotic pressure the fluorescence label was still detectable in all of the species we investigated and fluorescence readings were well above negative controls. Interestingly though for some species fluorescence readings of bacteria grown in the presence and absence of antibiotics were indistinguishable after overnight growth in liquid culture (Fig. 5). This finding may indicate the possibility to grow bacteria harboring the plasmid without antibiotic pressure for some time without loss of the fluorescence label. We would however advise to check this possibility for every species and every strain independently. Due to the heterogeneity we observed for plasmid loss in different species it does not seem possible to predict plasmid stability in the absence of antibiotics.

Due to the specific features of the plasmid we observed, high-level fluorescence, EGFP expression in very early logarithmic growth phase, and moderate plasmid stability despite the removal of antibiotic pressure, the reporter plasmid we constructed will probably be suitable for a wide variety of applications. It may be interesting for the investigation of bacterial invasion mechanisms, subcellular localization, phagocytosis, biofilm arrangement or intracellular survival. The usefulness of GFP and its derivatives for some of these applications has already been demonstrated in streptococci [18] [15] [27]. With our novel plasmid construct we hope to add to this knowledge by providing an easy to use general tool for many different bacterial species.
